# Mechanism of nimodipine in treating neurodegenerative diseases: in silico target identification and molecular dynamic simulation

**DOI:** 10.3389/fphar.2025.1549953

**Published:** 2025-03-13

**Authors:** Shuang Zheng, Yin Wang, Shuainan Tang, Yuntao Guo, Duan Ma, Xin Jiang

**Affiliations:** ^1^ School of Pharmacy, Anhui University of Chinese Medicine, Hefei, China; ^2^ Precision Genes Technology, INC., Nantong, China; ^3^ Yangtze Delta Drug Advanced Research Institute, Nantong, China

**Keywords:** nimodipine, calcium channel blocker, neurodegenerative disease, network analysis, molecular docking, molecular dynamics simulation

## Abstract

**Aim:**

Nimodipine has shown neuroprotective effects in several studies; however, the specific targets and mechanisms remain unclear. This study aims to explore the potential targets and mechanisms of nimodipine in the treatment of neurodegenerative diseases (NDDs), providing a theoretical foundation for repurposing nimodipine for NDDs.

**Methods:**

Drug-related targets were predicted using SwissTargetPrediction and integrated with results from CTD, GeneCards, and DrugBank. These targets were then cross-referenced with disease-related targets retrieved from CTD to identify overlapping targets. The intersecting targets were imported into STRING to construct a protein-protein interaction (PPI) network. Gene Ontology (GO) and Kyoto Encyclopedia of Genes and Genomes (KEGG) enrichment analyses were performed using the R package ClusterProfiler. Molecular docking was carried out using AutoDock Vina, and the ligand-receptor complexes with the highest binding affinities were further simulated using GROMACS to assess the dynamic structural stability and interactions between the ligand and receptor in the dynamic system.

**Results:**

A total of 33 intersecting drug-disease targets were identified. After constructing the PPI network and removing isolated targets, the network contained 28 nodes and 69 edges. Network degree analysis combined with enrichment analysis highlighted 12 key targets: CASP3, TNF, BAX, BCL2, IL1B, GSK3B, IL1A, MAOB, MAOA, BDNF, APP, and GFAP. Molecular docking analysis revealed binding energies greater than −6 kcal/mol for MAOA, GSK3B, MAOB, CASP3, BCL2, IL1B and APP. MAOA, with the highest binding energy of −7.343 kcal/mol, demonstrated a stable structure in a 100ns dynamic simulation with nimodipine, exhibiting an average dynamic binding energy of −52.39 ± 3.05 kcal/mol. The dynamic cross-correlation matrix (DCCM) of nimodipine resembled that of harmine, reducing the interactions between protein residues compared to the apo state (regardless of positive or negative correlations). Furthermore, nimodipine induced new negative correlations in residues 100-200 and 300-400.

**Conclusion:**

Nimodipine binds to the internal pocket of MAOA and shows potential inhibitory effects. Given its brain-enrichment characteristics and proven neuroprotective effects, it is hypothesized that nimodipine may exert therapeutic effects on NDDs by inhibiting MAOA activity and modulating cerebral oxidative stress. Thus, MAOA emerges as a promising new target for nimodipine in the treatment of NDDs.

## 1 Introduction

Neurodegenerative diseases (NDDs) refer to a group of disorders that damage the nervous system, leading to cognitive decline, memory loss, and motor dysfunction. The symptoms vary depending on the brain region affected (Wilson et al., 2023). Common NDDs include Amyotrophic Lateral Sclerosis (ALS), Multiple Sclerosis (MS), Parkinson’s Disease (PD), Alzheimer’s Disease (AD), and Huntington’s Disease (HD), with AD and PD being particularly prevalent (Erkkinen et al., 2018). As of 2019, AD affected an estimated 50 million people worldwide (Monfared et al., 2022), and this number is projected to rise to 150 million by 2050 (Lanctôt et al., 2024). This increasing prevalence poses significant challenges to the physical and mental wellbeing of the elderly (Hou et al., 2019), as well as to global healthcare systems.

The therapeutic strategies for NDDs primarily focus on the neurotransmitters and pathways associated with these diseases, aiming to restore abnormal factors to near-normal levels, though a complete cure remains elusive (Oskar, 2021). For example, PD is characterized by abnormal dopamine levels in the brain. Medications such as levodopa or carbidopa are used to replenish dopamine in the central nervous system, alleviating Parkinsonian symptoms (Armstrong and Okun, 2020). NDD patients are generally elderly, and many also suffer from cardiovascular diseases such as hypertension, coronary artery disease, and cerebral infarction, all of which are known risk factors for NDDs (Medina-Remón et al., 2018). These patients often require long-term administration of antihypertensive or anticoagulant medications. Clinical observations have noted that patients with both NDDs and cardiovascular diseases who are treated with calcium channel blockers (CCBs) experience alleviation of NDD symptoms, particularly in terms of reduced cognitive and motor impairments (Nimmrich and Eckert, 2013; Pasternak et al., 2012; Correa et al., 2023). However, the precise targets and mechanisms underlying these effects remain unclear. Some studies suggest that the vasodilatory effects of CCBs, which improve cerebral blood flow, may play a role. Other studies have demonstrated the neuroprotective effects of CCBs through both *in vivo* and *in vitro* experiments (Bork et al., 2015; Daschil and Humpel, 2014; Boltz et al., 2022; Kusakabe and Hasegawa, 2021; Leisz et al., 2019; Topcu et al., 2023). CCBs can be structurally classified into dihydropyridines, benzothiazepines, and phenylalkylamines, with verapamil and diltiazem representing the latter two classes. Notably, reports have suggested that these medications may induce Parkinsonian symptoms. Most research has focused on dihydropyridine CCBs. Despite belonging to the same class, these drugs differ significantly in their pharmacokinetic and pharmacodynamic properties due to structural variations. Some dihydropyridines predominantly exert peripheral effects, while others, which possess better lipophilicity, can cross the blood-brain barrier and act within the brain. From a mechanistic perspective, the latter group holds greater potential as therapeutic agents for NDDs (Colbourne and Harrison, 2022).

Nimodipine is considered the ideal candidate due to its superior permeability across the blood-brain barrier and its selective targeting of L-type calcium channels in cerebral blood vessels, promoting their relaxation. This property makes nimodipine an essential medication not only for the treatment of mild to moderate hypertension but also for the prevention and management of cerebral vasospasm, localized ischemia, and subarachnoid hemorrhage (Clough et al., 2022; Guangzhi et al., 2022; Carlson et al., 2020). In recent years, nimodipine has garnered attention as a potential neuroprotective agent, with its use in preventing and treating neurological dysfunction in patients with aneurysmal subarachnoid hemorrhage (aSAH) receiving approval from the US Food and Drug Administration (FDA). Additionally, studies have suggested that nimodipine may enhance cognitive function in patients with AD and PD (Hu et al., 2023; Lin et al., 2024; Ton et al., 2007), although the precise mechanisms underlying these effects have yet to be fully elucidated. Therefore, this study aims to investigate the potential targets and mechanisms of nimodipine in the treatment of neurodegenerative diseases using network analysis and molecular dynamics simulations.

## 2 Materials and methods

### 2.1 Screening of potential targets

The initial phase of the research involves querying and integrating drug and disease targets from various databases to identify the intersectional targets for subsequent analysis. Drug targets are identified through structural prediction methods and correlated with existing research findings. Disease targets are primarily selected based on experimental validation, with a focus on those implicated in disease progression or treatment.

The SMILES structure of nimodipine was obtained from the PubChem database (https://pubchem.ncbi.nlm.nih.gov) and input into the SwissTargetPrediction (Daina et al., 2019) platform (http://www.swisstargetprediction.ch/) for target prediction. Targets with a probability greater than zero were selected. Nimodipine-related targets were identified using the CTD (Davis et al., 2021) (https://ctdbase.org/), DrugBank (Knox et al., 2024) (https://go.drugbank.com/), and GeneCards (Stelzer et al., 2016) (https://www.genecards.org/) databases. From the GeneCards results, only targets with a “Relevance Score” above the median (0.475,787) were chosen. All identified targets were combined, deduplicated, and standardized using the UniProt (UniProt Consortium, 2025) database (https://www.uniprot.org/). In the CTD database, the following keywords were used for the search: Alzheimer’s Disease, Parkinson’s Disease, Huntington’s Disease, Amyotrophic Lateral Sclerosis, Dementia, Motor Skills Disorders, Cognitive Disorders, Learning Disabilities, and Amnesia. Only targets with direct evidence of mechanism and treatment were considered. The resulting targets were then integrated, deduplicated, and standardized with the UniProt database. Ultimately, the intersectional targets were identified as potential therapeutic targets for further investigation.

### 2.2 Network and enrichment analysis

The identified potential targets were imported into the STRING (Szklarczyk et al., 2023) database (https://string-db.org/) to construct a protein-protein interaction (PPI) network. The “Multi-protein Association Analysis” option was selected, with the species set to “*Homo sapiens*” and the confidence level set to 0.7. The network was exported to Cytoscape (Paul et al., 2003) (https://cytoscape.org/) for visualization, and the CytoHubba (Chin et al., 2014) package was used to calculate the network’s topological parameters. The Bioconductor R package ClusterProfiler (Yu et al., 2012) was utilized to perform GO and KEGG enrichment analysis on all potential targets, and the R package ggplot2 (Wickham, 2016) was employed to generate visualization charts and images of the enrichment results. For enhanced graphical presentation, the OmicShare platform (https://www.omicshare.com/) was used to refine the visuals.

### 2.3 Molecular docking

Significant targets identified through network and enrichment analyses were selected for molecular docking simulations. Protein models of these targets were retrieved from the Protein Data Bank (PDB) (https://www.rcsb.org/), while the structure file of nimodipine was sourced from the PubChem database. The preprocessing of proteins and ligands was conducted using a sequential approach. Initially, PrankWeb (Jendele et al., 2019) (https://prankweb.cz/) was employed to predict the binding pocket, which informed the adjustment of the grid box size and position. Subsequently, AutoDockTools (Forli et al., 2016) was used to add missing hydrogen atoms, calculate molecular charges, and finalize the grid box settings for the protein docking region. Following these steps, a configuration file for Vina was generated. AutoDock Vina (Eberhardt et al., 2021) was then employed to conduct 100 docking simulations, with the complex conformation exhibiting the most favorable binding energy being retained in PDB format. Discovery Studio Visualizer (Dassault Systèmes, 2024) was subsequently utilized to analyze the interactions between the ligand and receptor and to export visual representations of the results.

### 2.4 Molecular dynamic simulation

Previous complex with highest binding energy was then subjected to molecular dynamics simulations. The pdbqt file was imported into PYMOL (Odinger, 2024) software (https://pymol.org/) and converted into pdb format. The nimodipine molecule was pre-processed by adding missing hydrogen atoms, saved in mol2 format, and the molecule topology file was generated using Sobtop (Lu, 2024) software. Molecular dynamics simulations were subsequently carried out using GROMACS (James Abraham et al., 2015) 2024.4 software. The simulation system was set up in a dodecahedron box, utilizing the Amber99sb-ildn force field. All components were solvated in the TIP3P water model, and Na^+^ and Cl^−^ ions were added to neutralize the system charge.

The energy minimization (EM) phase used the steepest descent integrator, terminating when the maximum force dropped below 10.0 kJ/mol. For the NVT (constant number of particles, volume, and temperature) and NPT (constant number of particles, pressure, and temperature) equilibration phases, the velocity Verlet algorithm was employed as the integrator. Temperature coupling was managed using the V-rescale method, and pressure coupling was handled via the C-rescale method, with each phase undergoing an initial simulation duration of 100 ps. Notably, during the temperature coupling phase, the index groups Protein_MOL and Water_and_ions were fitted separately to avoid potential system instabilities. In the molecular dynamics (MD) phase, time step was set to 2 fs, and the total simulation duration spanned 100 ns. Trajectories were recorded every 10 ps, resulting in a dataset of 10,000 frames. The simulation was performed at a constant temperature of 300 K. Additionally, during the simulations, positional restraints of 1,000 kJ/mol/nm^2^ were applied to the ligand to preserve its configuration.

Structural analyses, including the calculation of root-mean-square deviation (RMSD) and root-mean-square fluctuation (RMSF), along with hydrogen bond analysis, were performed using the built-in commands of the GROMACS software suite. For calculating and visualizing the dynamic cross-correlation matrix (DCCM), the covar command was first used to generate eigenvectors. Subsequently, the gmx_corr script (https://github.com/busrasavas/gmx_corr) was employed for comprehensive analysis and figure generation. Binding energy assessment during the equilibrium phase was conducted with the gmx_MMPBSA (Valdés-Tresanco et al., 2021) tool, complemented by the gmx_MMPBSA_ana utility for visualization. All xvg files resulting from these procedures were visualized using the qtGrace software (https://sourceforge.net/projects/qtgrace/).

## 3 Results

### 3.1 Target screening

The results from all selected databases were integrated and deduplicated, yielding a total of 231 drug-related targets (Supplementary Table S1) and 332 disease-related targets (Supplementary Table S2). Among these, 33 intersection targets (Supplementary Table S3) were identified as potential therapeutic targets of nimodipine for NDDs and were selected for further analysis.

### 3.2 Network and enrichment analysis

**FIGURE 1:**
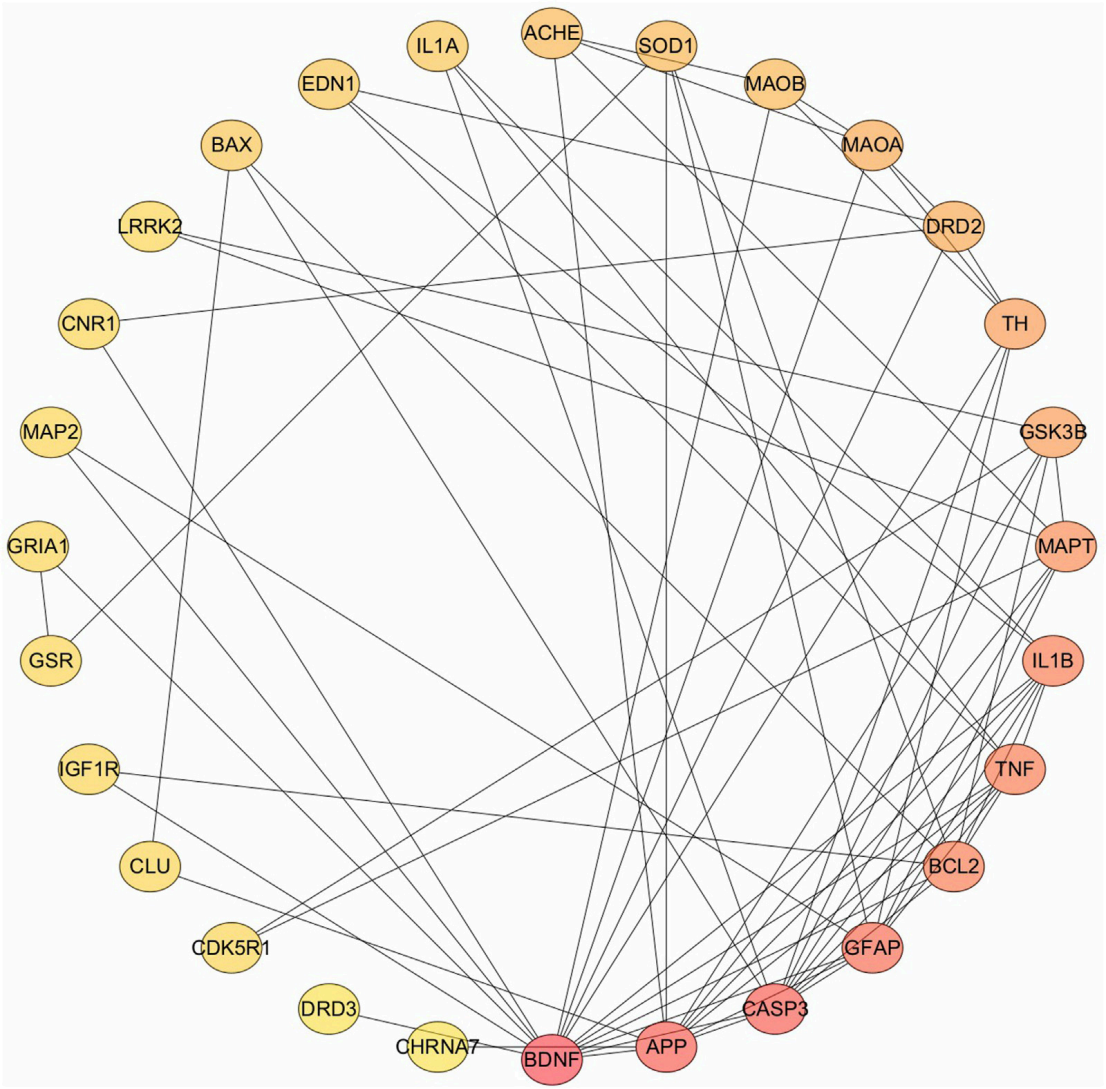
Protein-protein interaction network, rank by degree value.

The 33 potential targets were imported into the STRING database to construct the PPI network. After excluding five unconnected targets, the resultant network consisted of 28 nodes and 69 interaction edges, with an average node degree of 4.93 and an average local clustering coefficient of 0.535. These metrics indicate a strong interconnectivity among adjacent nodes, highlighting the network’s cohesive structure. The PPI enrichment P-value is extremely low (<10^−16^), underscoring the non-random nature of this protein interaction network, thereby affirming its biological relevance and the interrelation of the involved proteins. The targets were ranked according to their degree values (Figure 1), and the top five were selected for further investigation: (1) BDNF, (2) APP, (3) CASP3, (4) GFAP, and (5) BCL2. It is noteworthy that TNF and IL1B share the fifth rank in terms of degree value.

**FIGURE 2:**
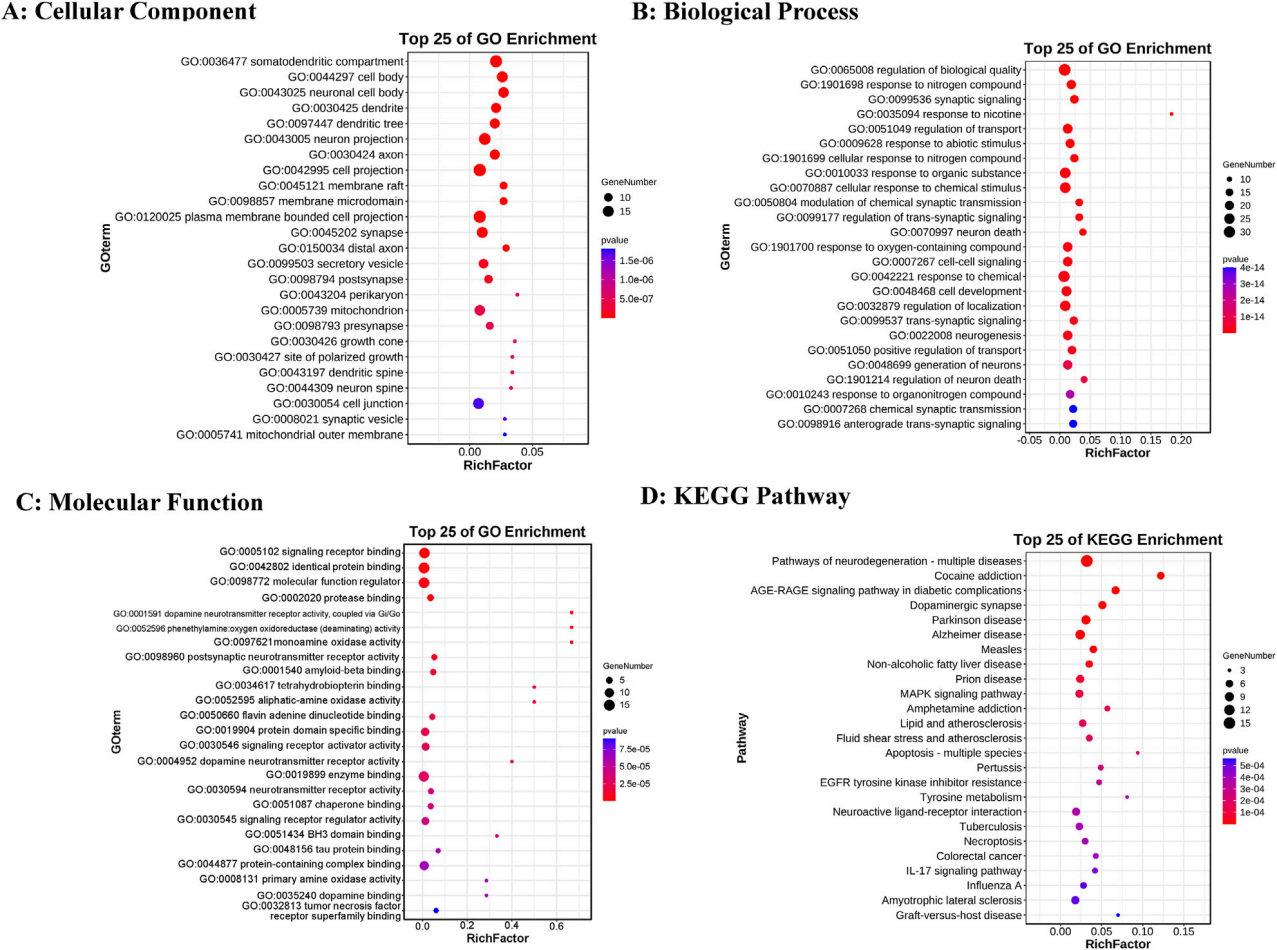
GO and KEGG enrichment analysis results. **(A)** Top 25 cellular components. **(B)** Top 25 biological processes. **(C)** Top 25 molecular functions. **(D)** Top 25 KEGG pathways. The color intensity represents varying p-value thresholds, while the dot size reflects the number of genes associated with each term.

Gene Ontology (GO) enrichment analysis results were categorized into three domains: cellular components, molecular functions, and biological processes. The top five annotations for cellular components (Figure 2A) are: soma-dendritic region; cell body; neuronal cell body; dendrite; and dendritic tree. The top five annotations for molecular functions (Figure 2B) are: signal receptor binding; homotypic protein binding; molecular function regulator; protease binding; and dopamine neurotransmitter receptor activity via Gi/Go coupling. The top five annotations for biological processes (Figure 2C) are: regulation of biological quality; response to nitrogen compounds; synaptic signaling; response to nicotine; and transport regulation. The top five KEGG enrichment results (Figure 2D) include: neurodegenerative disease pathways (multiple diseases); cocaine addiction; the role of AGE-RAGE signaling pathways in diabetic complications; dopaminergic synapses; and Parkinson’s disease.

**FIGURE 3:**
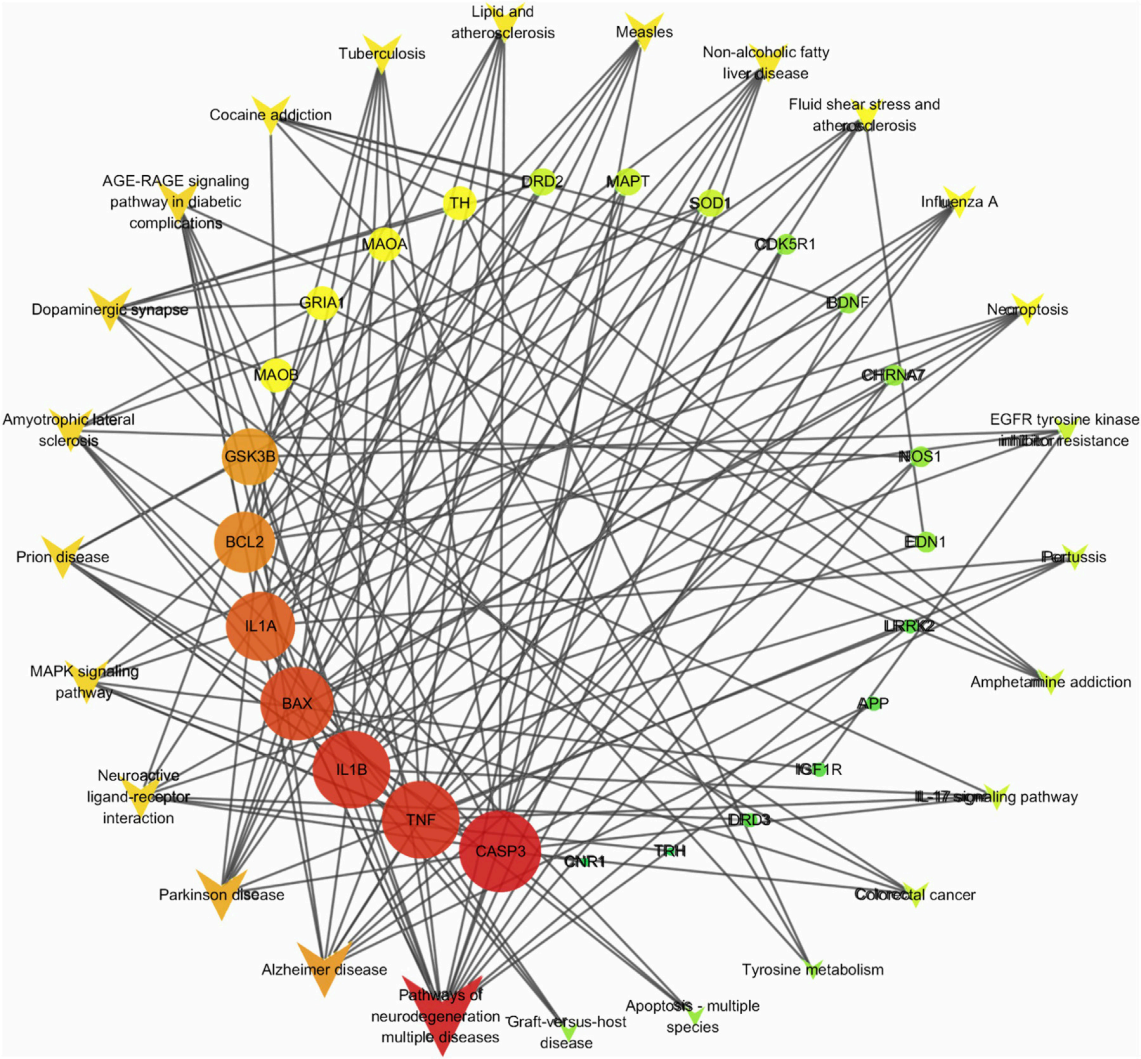
Target-pathway network. The quadrilateral represents the pathway, the circle represents the target, and the intensity of red indicates the degree value.

The KEGG enrichment analysis results (Supplementary Table S4) reveal that 29 targets are significantly enriched in 64 pathways, with 9 targets occurring frequently (>10 times), highlighting their pivotal role in the enriched pathways. These targets include: CASP3, TNF, BAX, BCL2, IL1B, GSK3B, IL1A, MAOB, and MAOA. Based on their functional significance, these proteins can be grouped into apoptosis-related, inflammation-related, and oxidative stress-related categories. Figure 3 illustrates the network interactions between the top 25 pathways and targets.

### 3.3 Molecular docking

The targets identified through the degree and enrichment analyses were integrated and deduplicated, resulting in a final list of 12 targets: CASP3, TNF, BAX, BCL2, IL1B, GSK3B, IL1A, MAOB, MAOA, BDNF, APP, and GFAP. These proteins were subjected to molecular docking with nimodipine to assess their binding affinity (Supplementary Table S5). Seven targets exhibited binding energies below −6 kcal/mol with nimodipine, indicating relatively stable binding. The targets were ranked by binding affinity as follows: MAOA, GSK3B, MAOB, CASP3, BCL2, IL1B, and APP.

**FIGURE 4:**
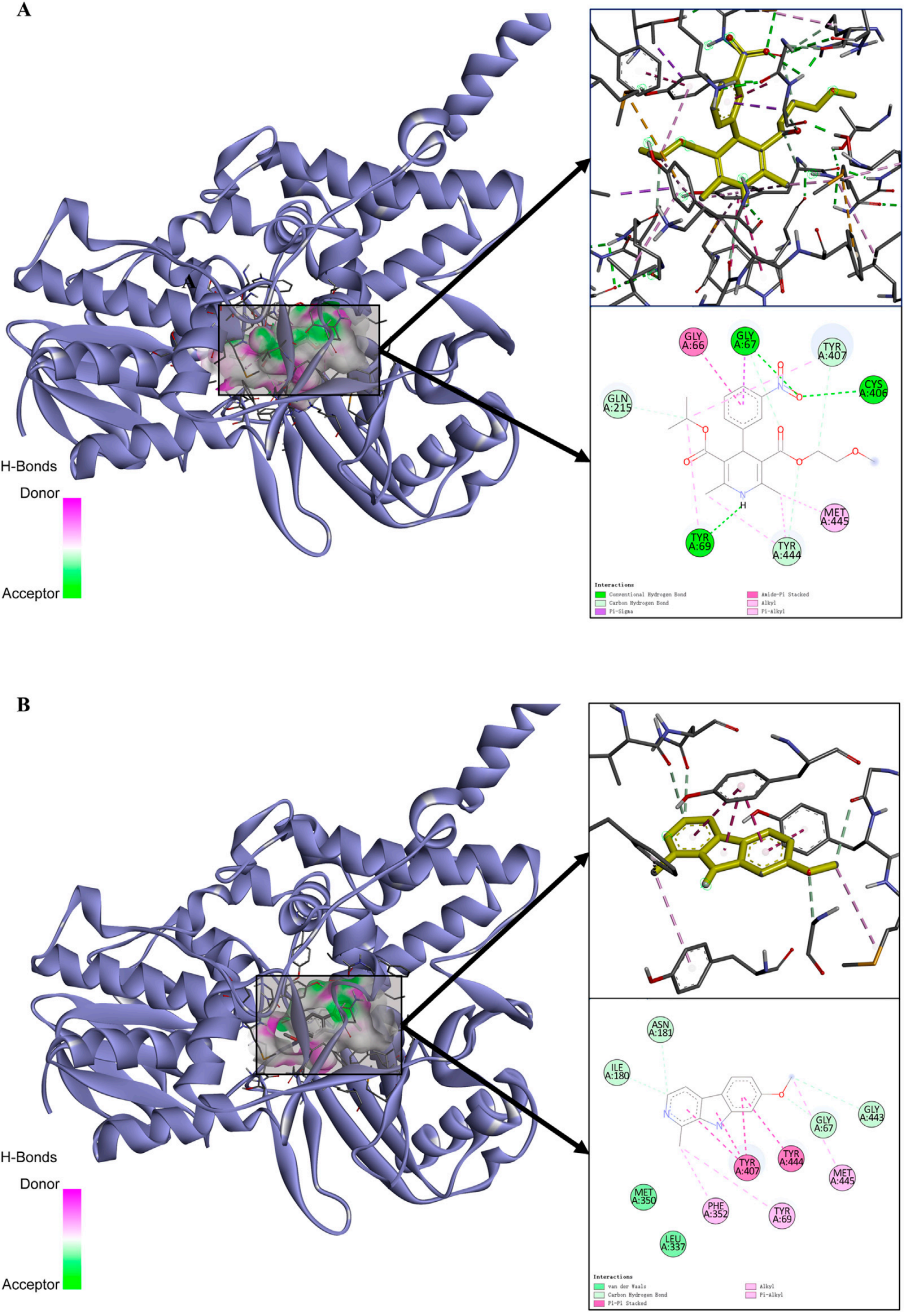
Interactions of MAOA with **(A)** nimodipine and **(B)** harmine, in 3D and 2D formats.

MAOA, with the highest binding energy, was selected for an in-depth analysis of its interactions. To compare the effect of nimodipine on MAOA, docking results of harmine, a known MAOA inhibitor with a binding energy of −8.476 kcal/mol, were used as a reference. The PDB model 2Z5Y inherently includes the small molecule harmine. Utilizing this ligand directly provides the best fit for the receptor model without inducing any adverse effects, ensuring reliable comparative results with nimodipine. Nimodipine’s interaction with MAOA is characterized as follows: the amino oxygen atom forms two hydrogen bonds with MAOA residues 67GLY and 406CYS; the nitrogen atom on the dihydropyridine ring forms a hydrogen bond with 69TYR; the amide ring engages in an amide-pi stacking interaction with 66GLY; and alkyl interactions, such as pi-sigma and pi-alkyl interactions, are also observed (Figure 4A). In contrast, harmine’s three rings form pi-pi bonds with 407TYR, while the benzene ring forms a pi-pi bond with 444TYR, resulting in a total of four pi-pi stacks. Additionally, some weak alkyl group interactions are noted (Figure 4B). According to relevant literature (Maurice Geha et al., 2002; Son et al., 2008), residues 305LYS, 397TRP, 407TYR, and 444TYR are functionally critical for MAOA. Residues 305LYS, 397TRP, and 407TYR are likely involved in non-covalent binding with FAD, while 407TYR and 444TYR may form aromatic stacking to stabilize substrate binding (similar to harmine). Although nimodipine does not directly bind to these residues in the static docking results, it forms spatial hindrances near these residues, which may influence their normal physiological functions.

### 3.4 Molecular dynamic simulation

#### 3.4.1 Structural analysis

**FIGURE 5:**
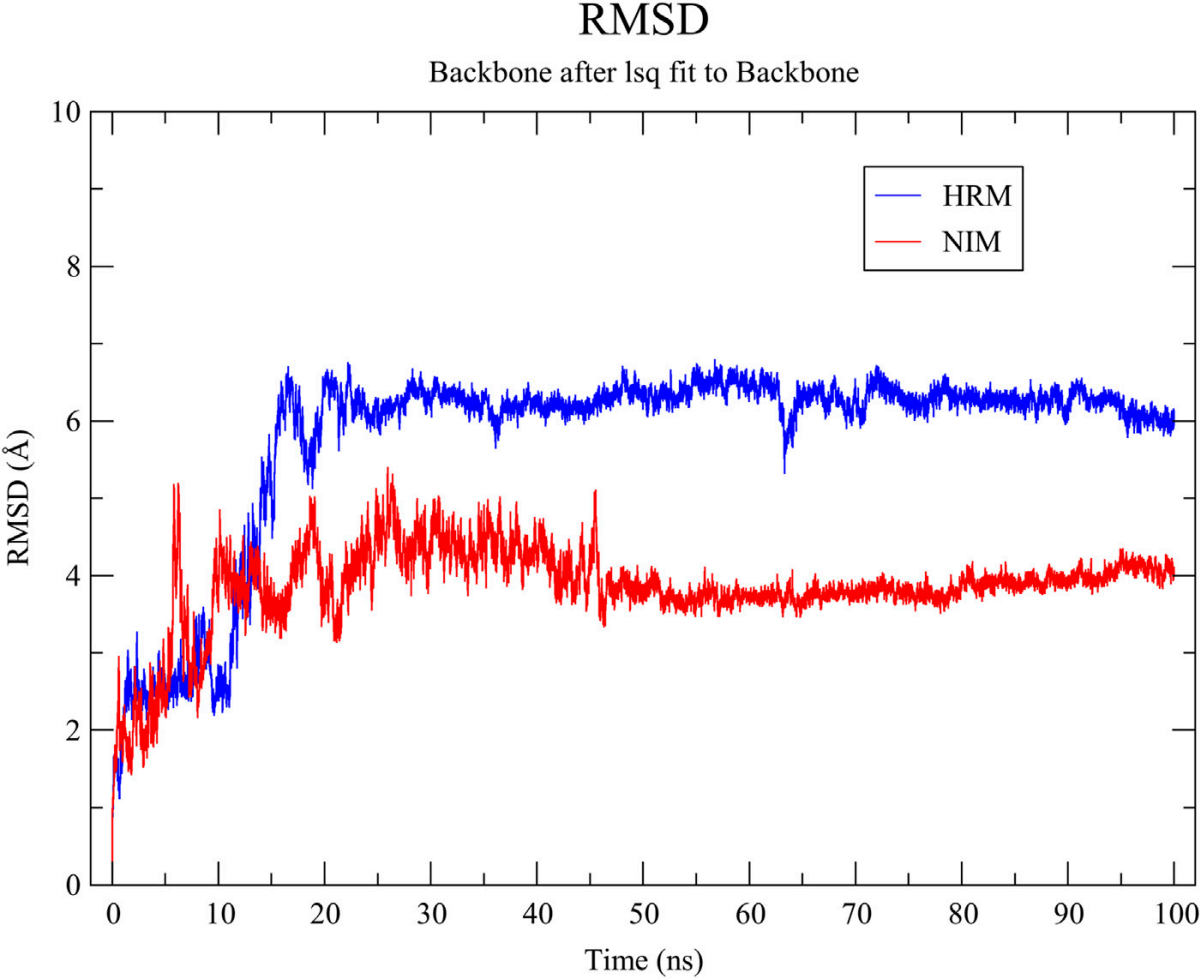
Root mean square deviation of the protein backbone of MAOA with nimodipine (NIM) and harmine (HRM).

**FIGURE 6:**
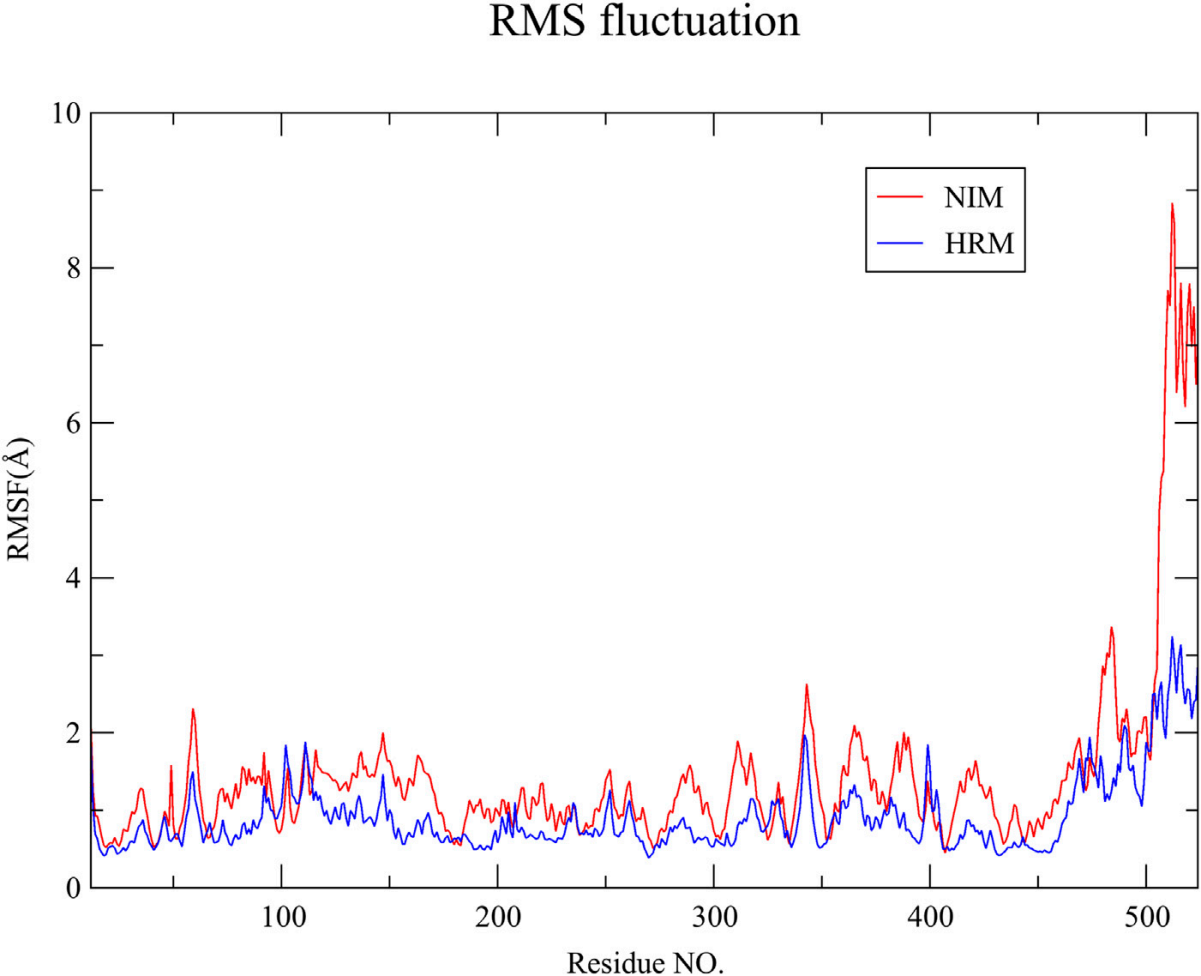
Root mean square fluctuation of MAOA Cα in the last 80 ns.

RMSD of the receptor’s backbone tends to reach stability after approximately 20 ns in the simulation with both ligands (Figure 5). Therefore, the last 80 ns of trajectories were selected for calculating the RMSF. As illustrated in Figure 6, the RMSF values of most Cα atoms in the MAOA-nimodipine complex are generally higher than those in the MAOA-harmine complex, indicating that these regions of the molecule exhibit greater mobility during the simulation. The residues after position 500 correspond to the tail of the protein, where fluctuations are more pronounced.

#### 3.4.2 Dynamic cross-correlation matrix

**FIGURE 7:**
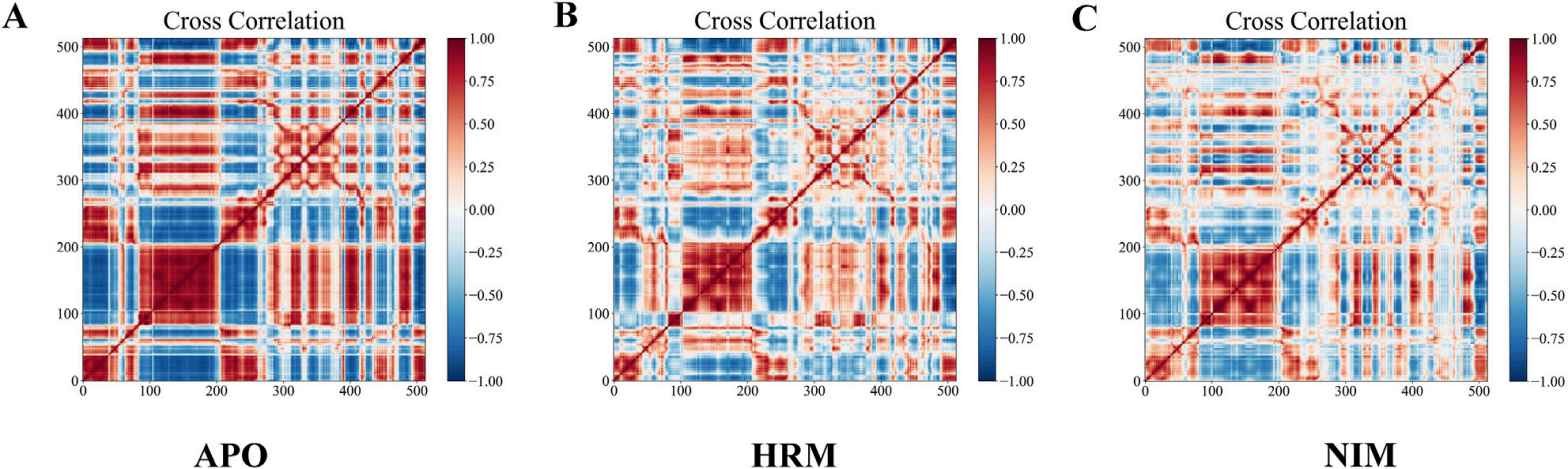
Dynamic cross-correlation matrix of Cα of MAOA in **(A)** unbound, **(B)** harmine-bound, and **(C)** nimodipine-bound states. Red indicates positive correlation, blue indicates negative correlation, and the intensity of color indicates the degree of correlation.

Figure 7 presents the DCCM of Cα atoms in MAOA for three different states: the APO state, bound to harmine, and bound to nimodipine. In these images, red indicates positive correlations, while blue indicates negative correlation. Harmine appears to weaken some correlations, both positive and negative, and can even convert positive correlations into negative correlations at specific positions. The effect of nimodipine is similar to that of harmine but more pronounced, with a stronger weakening effect. Specifically, nimodipine induces more new negative correlations, particularly in the NO.100-200 and NO.300-400 regions, suggesting a more significant alteration in the dynamic behavior of MAOA.

#### 3.4.3 Hydrogen bond analysis

**FIGURE 8:**
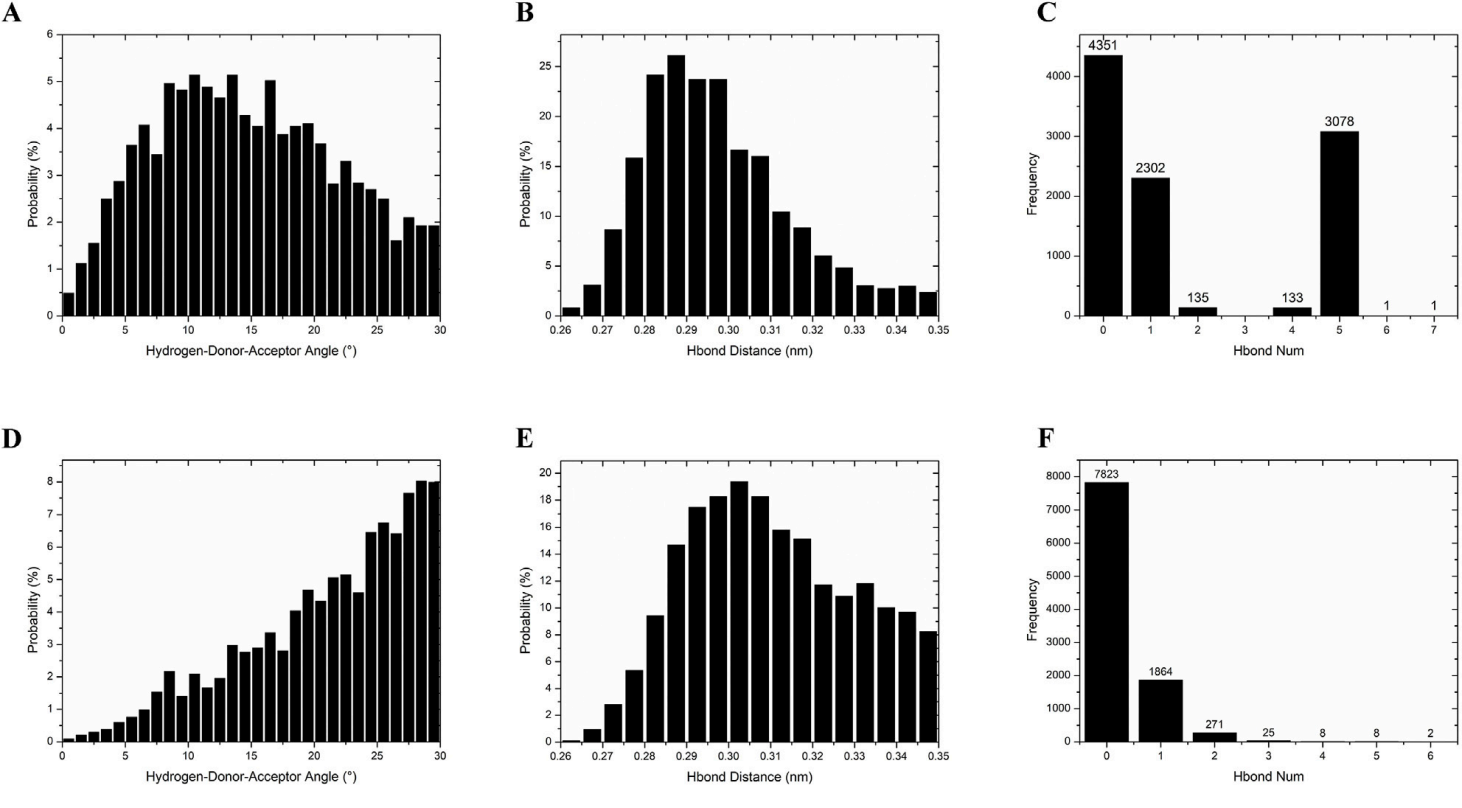
Hydrogen bond analysis of MAOA with harmine and nimodipine. **(A)** The probability of the angle for hydrogen bonding between harmine and MAOA. **(B)** The probability of hydrogen bonding distance between harmine and MAOA. **(C)** The frequency of hydrogen bonds number per frame between harmine and MAOA during the entire simulation. **(D)** The probability of the angle for hydrogen bonding between nimodipine and MAOA. **(E)** The probability of hydrogen bonding distance between nimodipine and MAOA. **(F)** The frequency of hydrogen bonds number per frame between nimodipine and MAOA during the entire simulation.

While the static molecular docking results indicated that nimodipine formed three hydrogen bonds with the protein, whereas harmine formed none, the dynamics simulation revealed a different scenario. In approximately 80% of the total 10,000 frames, nimodipine exhibited no hydrogen bonds (Figure 8F), while harmine maintained one or more hydrogen bonds in about 60% of the time frames (Figure 8C). Regarding hydrogen bond angles, nimodipine typically formed larger angles (Figure 8D), whereas harmine showed more consistent and average angles (Figure 8A). The distributions of hydrogen bond distances for both ligands were similar (Figures 8B, E), indicating that, despite differences in hydrogen bond formation and geometry, both ligands exhibited comparable distance characteristics in their interactions.

#### 3.4.4 Dynamic energy analysis

**FIGURE 9:**
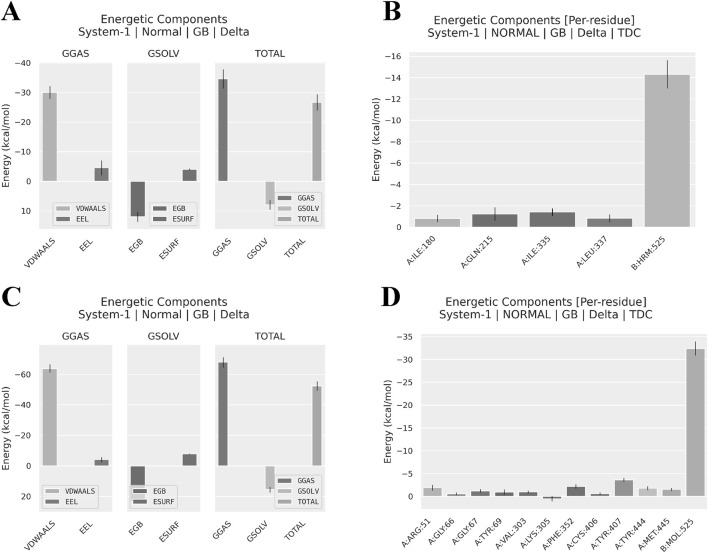
Software-predicted components of binding energy and residue contributions to binding energy. **(A)** Components of Harmine-MAOA binding energy. **(B)** Residue contributions to Harmine-MAOA binding energy, showing residues within 4Å. **(A)** Components of Nimodipine-MAOA binding energy. **(B)** Residue contributions to Nimodipine-MAOA binding energy, showing residues within 4Å. GGAS, Generalized Gradient Approximation for Solids; GSOL, Generalized Solid State Solvation Model; VDWAALS, Van der Waals forces; EEL, Electrostatic Energy; EGB, Electrostatic Energy of the Gas phase; ESURF, Electrostatic Energy of the Surface; TOTAL, total energy.

The analysis results (Figure 9; Table 1) reveal that nimodipine exhibits a higher binding energy (−52.39 ± 3.05 kcal/mol) with MAOA compared to harmine (−26.66 ± 2.75 kcal/mol), indicating a more stable association between nimodipine and MAOA during the simulation period. The key distinction between the two ligands lies in the van der Waals forces. Additionally, an analysis of the energy contributions of residues within a 4 Å radius indicates that nimodipine involves more residue interactions, including functionally critical residues of MAOA, such as 305LYS, 407TYR, and 444TYR, previously highlighted in the docking section. Although these residues do not directly bind to nimodipine in the docking conformation, interactions with nimodipine are observed during the dynamic simulation process.

**TABLE 1:** Dynamic energy analysis of MAOA with nimodipine and harmine.

Energy components	Nimodipine	Harmine
Van der Waals	−63.86 ± 2.73	−30.06 ± 2.16
Electrostatic	−4.13 ± 1.75	−4.55 ± 2.48
Polar salvation	23.47 ± 1.83	12.02 ± 1.66
SASA energy	−7.87 ± 0.20	−4.07 ± 0.24
Binding energy	−52.39 ± 3.05	−26.66 ± 2.75

Note: The unit of energy is kcal/mol.

## 4 Discussion

NDDs are frequently associated with inflammation in the central nervous system (CNS) (Chatterjee, 2016). Inflammatory factors encompass a broad and complex concept, as disruptions in multiple pathways can potentially trigger inflammatory responses (Zhang et al., 2023). The activity of monoamine oxidase A (MAOA) is capable of modulating the levels of inflammatory factors within the nervous system. In neuroinflammatory conditions, such as when microglia are activated by interleukin-1β (IL-1β), there is an induction of pro-inflammatory cytokine release, including tumor necrosis factor-α (TNF-α) and interleukin-6 (IL-6), along with an increase in the expression and activity of MAOA (Pallio et al., 2021). During the oxidative deamination of neurotransmitter amines, MAOA generates harmful byproducts such as ammonia, peroxides, and aldehydes. These substances can exacerbate oxidative stress and further damage neurons, thereby intensifying neuroinflammatory responses (Shoaib and Hoda, 2020). In Alzheimer’s disease, the activation of MAOA is directly correlated with altered concentrations of biochemical neurotransmitters in the brain. The oxidative stress it induces contributes to cholinergic neuronal damage and dysfunction of the cholinergic system, promoting the aggregation of neurofibrillary tangles and cognitive decline (Behl et al., 2021). MAOA plays a crucial role in neuronal apoptosis and autophagy, with its byproducts, such as hydrogen peroxide, generally inducing apoptosis. Additionally, the interaction between toxins and MAOA, leading to the opening of the mitochondrial permeability transition pore, is considered a potential mechanism of cell death. Inhibiting excessive MAOA activity can protect neurons from death (Lin et al., 2017).

Nimodipine is notable for its distinct pharmacokinetic profile and is primarily indicated for the prevention and treatment of delayed ischemic neurological disorders and other cerebrovascular conditions. Its mechanism of action involves enhancing cerebral blood flow by dilating small cerebral arteries, thereby offering therapeutic potential in a range of cerebrovascular diseases. Despite its efficacy, nimodipine’s clinical application is somewhat constrained by its low bioavailability, a challenge that has been addressed through the development of sustained-release formulations, such as enteric solid dispersions, to improve drug solubility. In comparison to other dihydropyridine calcium channel blockers (DHP-CCBs) like amlodipine and felodipine, which are primarily used for managing hypertension and coronary heart disease by targeting L-type calcium channels, nimodipine extends its effects beyond calcium channel blockade. It also enhances collateral blood flow and exerts direct anti-ischemic actions, providing additional neuroprotective benefits (Edraki et al., 2009). In contrast to non-dihydropyridine calcium channel blockers (NDHP-CCBs) like verapamil and diltiazem, which are employed in arrhythmia treatment by modulating cardiac electrophysiological properties and exerting negative chronotropic, dromotropic, and inotropic effects, DHP-CCBs, including nimodipine, are more selective in their arterial action and are preferred for treating volume-dependent hypertension and atherosclerosis (Triggle, 2007). Overall, nimodipine’s pharmacokinetic properties and its specific role in cerebrovascular protection underscore its unique position among calcium channel blockers. While its low bioavailability presents a limitation, this can be mitigated through formulation improvements, and its neuroprotective effects are especially significant in the prevention and treatment of delayed ischemic neurological disorders (Tomassoni et al., 2008).

Based on the static results of molecular docking, the binding of nimodipine to MAOA creates steric hindrance to the coenzyme riboflavin (FAD), which is crucial for activating MAOA. This suggests that nimodipine may inhibit MAOA activity, subsequently reducing oxidative stress. The DCCM further confirmed that the effect of nimodipine on MAOA closely resembles that of the inhibitor harmine. This similarity implies that nimodipine may share a comparable mechanism with harmine. Such findings are significant for understanding the therapeutic potential of nimodipine in neurodegenerative diseases, as they suggest that nimodipine could regulate MAOA enzymatic activity, thereby contributing to its neuroprotective effects. It is important to note, however, that the positive and negative correlations in the DCCM do not directly correlate to positive or negative biological effects; rather, the inference is drawn by comparing nimodipine with harmine, the known inhibitor.

Since the late 20th century, nimodipine has been studied for the treatment of mental illnesses and neurodegenerative diseases. Numerous clinical and experimental studies have shown that nimodipine, either alone or in combination with other treatments, can alleviate symptoms of neurodegenerative diseases. A meta-analysis (López-Arrieta and Birks, 2000) involving thousands of patients demonstrated that nimodipine provides short-term benefits for primary, vascular, and mixed dementia, with good tolerability and a low incidence of adverse reactions. Several clinical studies (Wang et al., 2019; Han et al., 2014; Hui and Zi, 2019; Cui and Tan, 2021; Wang et al., 2018) suggest that combining nimodipine with standard medications can more effectively alleviate cognitive impairment caused by various factors. Most studies have focused on symptom relief or the *in vitro* protective effects on neuronal cells, attributing these effects to the additional benefits of calcium channel blockade, while overlooking specific molecular mechanisms. Our study, grounded on the hypothesis that nimodipine may have target effects beyond L-type calcium channels, aims to discover new modes of action within the nervous system, thereby providing novel interpretative insights into its neuroprotective effects. More importantly, our research emphasizes molecular interactions, adopting a research approach that spans from the microscopic to the macroscopic. In the context of numerous studies identifying associations between targets, diseases, and drugs, we conducted docking and dynamic simulation studies on the key target MAOA. By utilizing existing computational techniques, we aim to elucidate the interaction modes and binding sites of nimodipine and compare them with harmine, an MAOA inhibitor, to infer the underlying mechanism of nimodipine’s action.

It is crucial to recognize that nimodipine primarily functions as a calcium channel blocker. While exploring its potential novel effects, one must not overlook its inherent side effects, particularly in elderly patients with neurodegenerative diseases (NDDs). Notably, nimodipine can induce hypotension, thereby increasing the risk of orthostatic hypotension, syncope, and falls in this vulnerable population (Das and Zito, 2025). Moreover, in certain cases, its use may even exacerbate NDD symptoms. Calcium influx plays a vital role in maintaining normal neuronal excitability and neurotransmitter release. By blocking calcium channels, nimodipine inhibits this influx, subsequently reducing neurotransmitter release and impairing inter-neuronal communication (Saghian and Wang, 2022). Under pathological conditions such as ischemia, trauma, and epilepsy, calcium channel blockers may alter neuronal excitability, rendering neurons more susceptible to excitatory amino acids like glutamate, potentially leading to excitotoxicity (Arundine and Tymianski, 2003). Furthermore, the blockade of voltage-gated calcium channels (VGCCs) can disrupt synaptic plasticity, a fundamental process for learning and memory formation. Such disruption may impair the encoding, consolidation, and retrieval of memories (Mansvelder et al., 2019).

Despite its potential side effects, nimodipine’s novel mechanisms of action in neurodegenerative diseases present promising therapeutic opportunities. Repositioning existing drugs to explore novel mechanisms of action represents a promising and valuable strategy in drug development (Bibhuti et al., 2022), and this approach can be well-supported by existing evidence in the case of nimodipine. Initially, nimodipine has been shown to alleviate mitochondrial dysfunction in mice, protecting them from 1-methyl-4-phenyl-1,2,3,6-tetrahydropyridine-induced PD (Singh et al., 2016). This finding suggests that nimodipine may reduce cellular calcium overload, a common pathological feature observed in neurodegenerative diseases. In AD models, nimodipine has been found to relax pericytes, improve cerebral blood flow, and reduce immune cell stalling and hypoxia, indicating that it may exert neuroprotective effects by enhancing cerebral blood flow and mitigating ischemic damage (Korte et al., 2024). Furthermore, our computational studies suggest that nimodipine can interact with MAOA, a key enzyme involved in the regulation of monoamine neurotransmitters, providing an additional mechanism through which nimodipine may exert its therapeutic effects. Together, these findings offer a theoretical foundation and valuable insights into the potential targets and mechanisms of nimodipine in the treatment of neurodegenerative diseases. However, further experimental validation of these targets and specific interactions is necessary in future studies to fully elucidate the therapeutic potential of nimodipine in this context.

## 5 Conclusion

Nimodipine has the potential to inhibit the activity of the MAOA enzyme and modulate cerebral oxidative stress. This effect, combined with previous findings related to calcium channel blockade, enhancement of cerebral blood flow, and modulation of neurotransmitter metabolism, collectively supports the multifaceted mechanisms through which nimodipine operates in NDDs. These findings highlight nimodipine’s potential as a therapeutic agent for NDDs. Accordingly, MAOA emerges as a promising novel target for nimodipine in the therapeutic management of these disorders.

## 6 Limitations

While this study has provided valuable insights into the potential mechanisms of nimodipine in NDDs through molecular docking and dynamics simulations, several limitations should be acknowledged. These include the reliance on computational methods, which require further experimental validation; the exclusive focus on MAOA, which may overlook other potential targets; and the relatively short simulation time, which might not fully capture the nuances of ligand-receptor interactions. Future research should aim to validate these computational findings through extensive *in vitro* and *in vivo* studies to assess the therapeutic efficacy and underlying mechanisms of nimodipine in various neurodegenerative disease models. Additionally, future studies should explore nimodipine’s comprehensive treatment effects in comorbid scenarios involving both cardiovascular and neurodegenerative conditions and employ high-throughput screening to identify adjunctive therapies that could enhance nimodipine’s neuroprotective effects.

## Data Availability

The original contributions presented in the study are included in the article/Supplementary Material, further inquiries can be directed to the corresponding authors.
